# Mapping Behavioral and Social Drivers of Influenza Vaccine Uptake in Older Adults: A Scoping Review

**DOI:** 10.3390/vaccines13060624

**Published:** 2025-06-10

**Authors:** Enming Zhang, Shuhui Shang, Yufei Xing, Jiasong Cui, Chen Pan, Holly Seale, Qiong Fang

**Affiliations:** 1 School of Nursing, Shanghai Jiao Tong University, 227 South Chongqing Rd., Huangpu District, Shanghai 200025, China; zhangenming@sjtu.edu.cn (E.Z.);; 2 School of Population Health, University of New South Wales, F25, Samuels Building, Samuel Terry Ave., Kensington, NSW 2033, Australia; 3 Department of Nursing, Ruijin Hospital, School of Medicine, Shanghai Jiao Tong University, 197 Ruijin 2nd Rd., Huangpu District, Shanghai 200001, China; 4 Department of Nursing, Renji Hospital, School of Medicine, Shanghai Jiao Tong University, 160 Pujian Rd., Pudong New District, Shanghai 200127, China

**Keywords:** influenza vaccines, vaccination, older adults, behavioral and social drivers, facilitators, barriers, scoping review

## Abstract

**Background/Objectives:** Influenza vaccination plays a crucial role in reducing morbidity and mortality among older adults; however, uptake remains suboptimal, particularly in the post-COVID-19 pandemic. In many settings, countries have not recovered their influenza vaccine coverage rates to the same level as pre-COVID. Therefore, this scoping review systematically identified the behavioral and social drivers (BeSD) influencing influenza vaccination among older adults using the BeSD framework. **Methods**: A systematic search across five databases included quantitative, qualitative, and mixed-methods studies involving individuals aged 60 years and older. Data were charted across four BeSD domains: thinking and feeling, social processes, motivation, and practical issues. **Results**: Thirty-nine studies from 24 countries were included. Key barriers encompassed safety concerns, misinformation, financial burdens, logistical challenges, and cultural and language barriers. While motivation was positively associated with vaccination intentions, the transition from intention to behavior remains underexplored, and practical issues have received comparatively limited research attention. **Conclusions**: These findings underscore the need for multifaceted, behaviorally informed interventions and greater inclusion of under-resourced settings to support equitable influenza vaccination strategies for healthy aging.

## 1. Introduction

Seasonal influenza continues to pose a significant global public health issue, causing an estimated 290,000–650,000 respiratory deaths each year [1]. Individuals aged 60 years and older are particularly vulnerable due to immunosenescence and an increased incidence of chronic comorbidities [2,3]. The World Health Organization (WHO) recommends annual influenza vaccination for the elderly as a key preventative strategy in consideration of these hazards [4]. Despite established immunization initiatives and strong public health guidelines, influenza vaccine uptake among older adults consistently falls short in many regions. Vaccination rates in countries belonging to the Organization for Economic Co-operation and Development (OECD) average around 55% [5], considerably lower than the WHO’s target of 75% coverage [4]. This persistent shortfall indicates that improving supply and access alone is insufficient, as behavioral and social determinants are crucial in influencing vaccination [6]. Although conventional strategies have focused on improving vaccine supply, accessibility, and policy implementation, these efforts have proven inadequate in bridging the immunization disparity among the elderly [7,8].

In response to the persistent poor coverage, research attention has increasingly shifted toward addressing individual-level factors, such as personal beliefs, risk perceptions, and psychological attitudes toward vaccination [9,10,11]. However, there has been more limited focus on the roles of relationships, community dynamics, intrinsic motivation, and practical or logistical barriers—which may be vital in shaping vaccination behavior among older adults [12]. A thorough comprehension that synthesizes these multi-tiered factors is crucial for developing more intervention strategies. In addition, the COVID-19 pandemic has further complicated this landscape by transforming public attitudes toward vaccines, modifying healthy lifestyles, and disrupting influenza transmission patterns [13].

In 2022, the WHO introduced the Behavioral and Social Drivers (BeSD) framework to help address these challenges. This framework integrates vaccination drivers into four interrelated domains: thinking and feeling, social processes, motivation, and practical issues [14]. The BeSD serves as a holistic approach, bridging gaps left by previous research that mainly emphasized structural or socioeconomic determinants [15]. However, there is a deficiency of evidence syntheses employing the BeSD framework that concentrates on vaccinations for the elderly, particularly those that ensure equitable attention across all domains [10,15,16,17].

This scoping review aims to systematically apply the BeSD framework to map and integrate current research about behavioral and social determinants influencing influenza vaccine uptake among older adults globally. This review intends to improve the health outcomes and vaccination coverage of geriatric populations by integrating findings across cognitive, social, motivational, and practical dimensions, thereby informing public health policymaking and guiding the development of behaviorally informed strategies.

## 2. Materials and Methods

This scoping review was executed following the Joanna Briggs Institute (JBI) methodology and reported following the Preferred Reporting Items for Systematic Reviews and Meta-Analyses extension for Scoping Reviews (PRISMA-ScR) [18]. A protocol for this review has been developed, and the PRISMA-ScR checklist is included in Appendix A: Appendix A.1. and Appendix A.2..

### 2.1. Conceptual Framework

This review was conducted using the BeSD framework, which is grouped and assessed across four domains: (i) thinking and feeling about vaccines, including beliefs, perceptions of disease risk, and vaccine confidence; (ii) social processes that drive or inhibit vaccination, including social norms, healthcare worker recommendations, and community-level influences; (iii) motivation or hesitancy in seeking vaccination; and (iv) practical issues related to obtaining and receiving vaccination, such as logistical and system-level barriers.

### 2.2. Research Questions

This scoping review seeks to explore the following three questions:

(i) What behavioral and social drivers influence influenza vaccine uptake among those aged 60 years and older?
(ii) What facilitators and barriers to influenza vaccination among older adults have been considered through the four domains of the BeSD framework?
(iii) What knowledge deficiencies exist in the current research addressing this population’s behavioral and social drivers of influenza vaccination?

### 2.3. Search Strategy and Selection Criteria

A thorough literature search was conducted in MEDLINE (via PubMed), EMBASE, Web of Science, CINAHL, and the Cochrane Library from inception to 31 December 2024. Principal search terms included influenza, vaccination, and older adults, along with terms associated with behavioral and social drivers (e.g., perception, acceptance, motivation, willingness). The terms were combined using Boolean operators and adapted to the specific search criteria of each database. The comprehensive search strategy is detailed in Appendix A: Appendix A.3.

Study eligibility was established by the PCC (Population, Concept, Context) framework [19]. The population (P) comprises individuals aged 60 years and older; concept (C) focuses on behavioral and social drivers of influenza vaccination with the BeSD framework, and context (C) requires grouping facilitators and barriers to influenza vaccination within this population with the BeSD framework. We included peer-reviewed publications that (i) concentrated on influenza vaccination in older individuals (aged 60 years or older), (ii) examined at least one domain of the BeSD framework, and (iii) were published in English without constraints on geographic location or research environment.

Articles were excluded if they: (i) concentrated on vaccine characteristics such as efficacy, safety, and immunogenicity; (ii) were restricted to cost-effectiveness, modeling, or budget impact assessments; (iii) focused on vaccines unrelated to the review questions; (iv) were intervention studies or secondary analyses derived from national surveys or extensive datasets. We additionally excluded specific types of publications, including clinical reports, guidelines, position papers, study protocols, book chapters, conference abstracts, editorials, duplicate studies, and studies lacking full text.

The primary outcomes of interest included (i) vaccine acceptance, refusal, delay, and consent; (ii) thinking and feeling: disease risk, vaccine confidence, perceived benefits, safety, trust, attitudes, beliefs, knowledge, and awareness; (iii) social processes: norms, healthcare provider recommendations, gender equity, and misinformation; (iv) motivation: intention, readiness, willingness, and hesitancy; and (v) practical issues: availability, convenience, cost, service quality, and barriers to access.

### 2.4. Study Selection

The retrieved literature was imported into Endnote 21 software (Clarivate, Philadelphia, PA, USA) for the management of references and the initial screening of articles. A meeting was convened to discuss and familiarize the research team with the eligibility criteria, and 20 randomly selected titles/abstracts were reviewed to detect discrepancies. The titles and abstracts were independently reviewed by two reviewers (S.S. and Y.X.) according to the established eligibility criteria. The full-text records of the articles that met the eligibility criteria were retrieved, screened, and extracted. The third reviewer (E.Z.) addressed all differences identified throughout the screening process, resulting in a consensus for all determinations.

### 2.5. Quality Appraisal

The methodological quality of all included studies was further evaluated according to their study design. We applied the proper versions of the JBI Critical Appraisal Checklists [20] for quantitative analytical cross-sectional studies and qualitative studies. Every item on the checklist was evaluated as “Yes”, “No”, “Unclear”, or “Not applicable.” Articles without “No” or “Unclear” ratings were categorized as strong quality. Articles receiving one to three “No” or “Unclear” scores were categorized as moderately strong, but those over three were considered poor. We employed the Mixed Methods Appraisal Tool (MMAT) 2018 version [21] to assess the methodological quality of mixed methods studies (MMS). Screening questions were initially used to verify study eligibility, followed by assessment against five specific criteria related to the integration and quality of qualitative and quantitative components. Every item was evaluated as “Yes”, “No”, or “Cannot tell”, and no overall score was calculated by MMAT guidelines. A comprehensive summary of the quality appraisal is provided in Appendix A: Appendix A.4.

### 2.6. Data Extraction, Charting, and Analysis

A draft information extraction form was developed following the research questions and tested on a sample of 10 included articles by two independent reviewers (S.S. and Y.X.). Following a research group meeting, the form was revised and finalized. We extracted basic study characteristics, including authors, year of publication, study settings, study design, study population and sample size, and study timeline. With the BeSD framework, we charted findings across four key domains: thinking and feeling, social processes, motivation, and practical issues. We additionally gathered data on relevant theoretical models, sociodemographic characteristics, health-related variables, and outcomes. All extracted data were documented in a standardized charting form, and findings were thematically synthesized to identify frequently reported facilitators and barriers within the BeSD domains.

## 3. Results

### 3.1. Selection of Sources of Evidence

**Figure 1:**
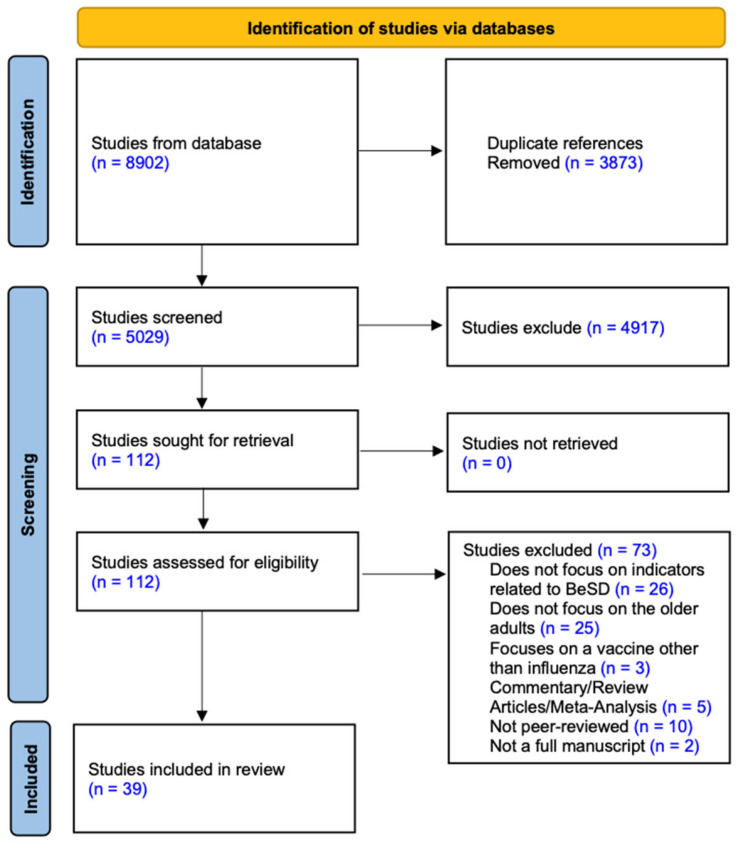
Screening and selection process.

Initial search results yielded 8902 records across all databases. After removing 3873 duplicates, 5029 records remained for title and abstract screening. Based on the eligibility criteria, 112 full-text articles were reviewed, of which 73 were excluded due to irrelevant outcomes, ineligible populations, or insufficient empirical data. Ultimately, 39 studies were included in the final data extraction and synthesis. All included studies underwent quality appraisal using standardized tools appropriate to their study design. Only studies rated as having moderate or high methodological quality were included in the synthesis. The selection process is illustrated in the PRISMA flow diagram (Figure 1).

### 3.2. Characteristics of Included Studies

The 39 included studies were conducted across 24 countries, with the largest number from China (including Hong Kong, *n* = 14) and the United States (*n* = 5), followed by studies from Europe and Asia. Most studies employed cross-sectional survey designs (*n* = 32), with four using qualitative methods and three employing mixed-methods approaches. Eighteen studies were conducted in high-income countries (HICs), 19 in middle-income countries (MICs), and two studies included settings in both HICs and MICs. No study focused exclusively on low-income countries (LICs). Sample sizes ranged from 10 to over 700,000 participants, and all the studies focused on adults aged 60 years or older. Most of the studies appeared to involve community-dwelling older adults, i.e., individuals not living in institutional care settings. However, not all studies explicitly reported participants’ residential environment; therefore, this variable was not systematically categorized in Table 1. The characteristics of the complete study are presented in Table 1.

Eleven studies applied theoretical models, including the Health Belief Model, the Theory of Planned Behavior, and the 3Cs Model. However, no clear pattern was observed in the model application based solely on the country’s income level, as studies from both high-income and middle-income countries employed similar frameworks. Sociodemographic variables commonly assessed included age, gender, education level, income, living status, residence type, and family structure. Health-related factors included comorbidities, chronic diseases, self-rated health, medication use, obesity, and vaccination history. The primary outcomes assessed were influenza vaccination behaviors and intentions. Theoretical frameworks, sociodemographic characteristics, health-related variables, and outcomes are summarized in Table 2.

### 3.3. Summary of Behavioral and Social Drivers by BeSD Domains

Findings were synthesized according to the four domains of the WHO BeSD framework. We identified common facilitators and barriers influencing influenza vaccination among older adults within each domain and summarized them below.

#### 3.3.1. Thinking and Feeling

Evidence from 39 studies demonstrated the significant influence of cognitive perceptions and emotional responses on vaccination behaviors among older adults. Key facilitating factors included the perceived risk of influenza infection—encompassing both susceptibility and severity—reported in 14 studies [9,22,24,25,27,37,38,40,41,43,46,53,56,57]. Positive vaccine attitudes, reflecting recognition of protective health benefits, were noted in seven studies [22,23,27,34,38,39,56]. Additionally, normative beliefs that supported vaccination behavior were identified in one study [23]. Vaccine confidence, particularly trust in the safety and efficacy of vaccines, was consistently associated with higher acceptance across 11 studies [29,32,36,42,45,46,49,53,54,55,57]. Furthermore, adequate vaccine literacy was linked to informed decision-making in six studies [45,46,48,49,52,54].

In contrast, concerns regarding vaccine safety and potential side effects were the most frequently reported barriers, as documented in 11 studies [22,27,31,33,37,41,43,45,49,53,57]. Procedural anxieties, such as fear of injections, were reported in two studies [8,57]. Additional barriers included low perceived susceptibility [33,45,55], negative vaccine beliefs and environmental concerns [50], limited vaccine literacy [44,56], cognitive overload [48], decisional conflict [48], and perceived cost concerns [56].

#### 3.3.2. Social Processes

This domain was explored in 24 studies, emphasizing the important role of interpersonal relationships, social dynamics, and community trust in influencing influenza vaccination behaviors among older adults. The most frequently reported facilitator within this domain was recommendations from healthcare workers (HCWs), identified in 17 studies [24,25,27,29,32,33,38,39,41,42,43,45,47,57,58]. Family influence was also reported in eight studies [30,33,34,38,45,57,58], where advice, emotional support, and encouragement from immediate family members were associated with an increased likelihood of vaccine uptake. Peer influence and recommendation—through informal discussions and social encouragement within friendship networks—were noted in four studies [33,34,57,58] as modest contributors to positive attitudes toward vaccination, although less prominently than HCWs or family recommendations. Broader social factors, such as social trust and community engagement, were reported as facilitators in six studies [26,36,41,52,56,57].

Despite the positive influences, researchers also identified social barriers. An absence of proactive HCW recommendations and negative healthcare interactions, such as insufficient information provision or lack of personalized counseling, was reported in two studies [8,50]. Additionally, the spread of misinformation within social networks and communities, highlighted in two studies [36,50], led to confusion, reduced trust, and increased hesitancy toward vaccination.

#### 3.3.3. Motivation

Ten studies addressed this domain, focusing on vaccination intentions and intrinsic motivational drivers. Clear vaccination intentions, reflecting a proactive willingness or plan to receive vaccines, were identified as key facilitators in five studies [24,30,34,51,57]. These studies indicated that individuals expressing definite intentions were more likely to receive vaccines. One study noted that the COVID-19 pandemic context further intensified such intentions [51]. Collective responsibility—characterized by moral motivations to protect family members and vulnerable groups—was reported in three studies [9,37,57]. This sense of social duty was particularly noted during heightened public health concerns, such as the COVID-19 pandemic, where protecting others was cited as a motivating factor. In contrast, vaccine hesitancy emerged as a significant barrier in three studies [36,53,56], reflecting doubts and resistance that hindered vaccination uptake.

#### 3.3.4. Practical Issues

Fifteen studies addressed practical issues related to access, availability, and service delivery that either facilitated or impeded vaccination uptake. Key facilitators included the availability of incentive-based vaccination programs, such as free or low-cost services, reported in six studies [28,41,43,46,51,53]. Service convenience, such as accessible vaccination sites, flexible appointment scheduling, and clear dissemination of vaccination information, was reported in two studies as further encouraging uptake [39,52].

Despite the presence of these facilitators, researchers identified several practical barriers. Financial constraints related to insufficient insurance coverage, high vaccine costs, or lack of subsidies were reported in three studies [8,37,57]. Accessibility challenges, such as transportation difficulties, limited clinic hours, and geographic inaccessibility, were cited in five studies [8,25,42,55,57], particularly affecting rural or underserved populations. Cultural and language barriers were reported in two studies [25,28], reflecting challenges experienced by minority groups or individuals with limited proficiency in the primary language of healthcare providers. Health system limitations included vaccine shortages, low service quality, and limited access to reliable information, as noted in three studies [25,48,57]. One study also identified postvaccination discomfort as a real-world obstacle to vaccination among older adults [40].

## 4. Discussion

This scoping review systematically applied the WHO BeSD framework to map behavioral and social determinants influencing influenza vaccination uptake in older adults globally. In accordance with previous research, the “thinking and feeling” dimension significantly influenced influenza vaccination behaviors in older persons, especially with vaccine confidence and risk perceptions. The confidence of the influenza vaccine’s safety and efficacy is identified as a crucial element contributing to vaccination efforts [59,60,61]. Risk perception content differed across various contexts and demographics. During the COVID-19 epidemic, there was a significant increase in public perceptions of the risk of infection [62,63]. Conversely, in the non-epidemic period, older individuals exhibited more significant anxiety regarding their susceptibility to influenza, whereas younger individuals prioritized the efficacy of vaccination [43]. While older people often exhibit less concern regarding vaccine side effects compared to younger adults [43], the misconception that influenza constitutes a “mild illness” and lingering fears about adverse effects persist as significant barriers to vaccinations among the elderly [64]. Furthermore, around 25% of older persons identified fear of needles as an impediment to vaccinations [8], indicating that emotional aspects require consideration.

Social processes were identified as a crucial domain influencing vaccination among older people. Beyond the well-established impact of HCWs’ recommendations, family and peer influences also acted as important social drivers of vaccine acceptance. Notably, older adults received vaccination recommendations from HCWs considerably more often than younger adults [43], indicating that health education strategies may be further refined according to this demographic’s characteristics to improve vaccination promotion. Emotional support, encouragement, and the modeling of positive health behaviors by family members were shown to enhance individuals’ willingness to vaccinate [65]. While typically less influential than familial influence, peer networks within communities contributed to positive vaccination attitudes through mechanisms such as reinforcement of social norms and the exchange of information [66]. However, the spread of misinformation through peer groups and online social networks significantly undermined trust in vaccines and promoted hesitancy [67,68]. These findings underscore the necessity for future interventions to transcend the individual level by enhancing community vaccine communication and refining HCWs’ communication skills to effectively rectify misinformation, promote trust, and cultivate an environment supportive of vaccination.

Practical issues considerably influence influenza vaccination in older persons, frequently surpassing attitudinal or motivational aspects. Free or low-cost immunization initiatives have proven an enhancement in accessibility to the influenza vaccine. A free vaccination program for the elderly in Beijing, China, has resulted in a significantly higher vaccination rate among the elderly (48.7%) compared to young individuals who must pay for their vaccines (16.0%) [43]. The structure of the healthcare system, particularly the coverage of universal healthcare, additionally establishes financial obstacles. The United States is a high-income country without universal health insurance coverage; studies have revealed that “high costs” [8] and “lack of insurance” [25] are significant barriers to access. Conversely, in HICs with universal healthcare, direct financial obstacles are less common, and providing free influenza vaccines in Australia has significantly increased vaccination rates [28]. In Singapore, despite plans such as Medisave that provide coverage for vaccinations, the uptake of vaccinations by older persons remains highly dependent on complete subsidization [37]. In MICs like China, while “free costs” frequently serve a positive function [43,46,51,53], issues of access and inconsistent coverage caused by the exclusion of influenza vaccines from the National Immunization Program (NIP) persist as obstacles [69,70]. Besides direct expenses, well-structured financial incentives have been shown to be beneficial in enhancing participation rates, especially among low-income populations and low- or middle-income countries (LMICs) with inadequate insurance coverage [71,72]. An innovative pilot project in China called “pay-it-forward” offers free vaccines and promotes donations, resulting in a threefold increase in vaccination rates among the elderly compared to the conventional fee-for-service model while simultaneously enhancing community engagement and confidence in vaccines [73], highlighting the potential of prosocial financing to address both economic and other behavioral determinants. However, insufficient awareness of funding schemes further constrains access to immunizations. In Poland, approximately 51.8% of older persons polled were cognizant of the 50% vaccine reimbursement program [35]. Despite financial obstacles, supply-side limitations, including vaccine shortages, posed substantial challenges to immunization efforts, particularly among marginalized groups [74,75,76]. Logistical challenges, including transportation difficulties, geographic isolation, and limited clinic hours, further restricted vaccine accessibility. These findings clarify the necessity for comprehensive, system-level interventions to address economic and logistical barriers. Recommended efforts include expanding funding mechanisms, ensuring stable vaccine supply chains, and improving geographic accessibility of appropriate immunization services.

While the BeSD framework provides a valuable structure, this review also identified important cultural factors associated with influenza vaccination in older adults. These aspects are profoundly embedded in cultural background, traditional values, and identity yet remain inadequately represented by the four established domains. Cultural and language barriers significantly contribute to vaccination inequities, particularly within minority communities [77,78,79]. For example, culturally tailored recommendations have been shown to improve uptake among groups such as elderly Haitians [25]. Similarly, individuals from non-English-speaking backgrounds often face reduced access to immunization services because language and cultural mismatches prevent effective communication with HCWs [28]. Culturally embedded health beliefs also play a crucial role. A preference for “natural immunization” is correlated with reduced vaccination rates [9,57]. Furthermore, cultural associations, prejudices regarding healthcare institutions, and provider attitudes contribute to diminished vaccine acceptability [50], underscoring the need to incorporate these features into promotional efforts. A multinational study highlighted that the prevailing culture affects vaccine accessibility and recommended incorporating indigenous health practices into immunization initiatives [57]. Collectively, these findings emphasize the necessity of integrating vaccination efforts with the cultural context, values, and experiences of the target population. We propose “Cultural Alignment” as a fifth domain of BeSD, a component inadequately underlined in the existing BeSD domains. Considering cultural alignment as an independent area will improve the cross-cultural relevance of the BeSD model, particularly in multicultural communities and LMICs [80,81,82]. It provides a realistic foundation for developing culturally sensitive interventions, including the personalization of health communications, the involvement of trusted community information, and the adaptation of services to align with traditional values, thereby overcoming critical conceptual deficiencies and advancing global equity in vaccinations.

It is noticeable that the COVID-19 pandemic has significantly altered the attitudes and behaviors of older adults regarding influenza vaccination, and research has highlighted the complex and sometimes contradictory implications. Initially, the willingness to receive influenza vaccination was heightened by increased awareness of respiratory viruses prior to the delivery of the COVID-19 vaccine; a Shanghai study reported that 68.4% of older adults expressed such intent [51]. However, this ideal trend was complicated by contradictory behaviors: individuals with a heightened concern about contracting COVID-19 were occasionally more inclined to avoid influenza vaccination (OR = 1.65, 95% CI [1.02–2.66]), potentially due to concern regarding healthcare exposure or an overwhelming emphasis on COVID-19 prevention [35]. The indirect consequences of COVID-19 mitigation strategies and vaccine advocacy introduce further complexity. Public health actions, such as mask-wearing, may reduce the risk of influenza and decrease the demand for vaccination [37]. Moreover, although prior COVID-19 vaccinations have been positively correlated with influenza vaccination [54], they have also been accompanied by misconceptions, particularly the belief that the COVID-19 vaccine offers cross-protection against influenza (“one shot is enough”), which has hindered vaccination efforts [37]. In addition, the ongoing emphasis on COVID-19 information may overshadow influenza-specific communications, diminishing awareness of influenza risk and the need for vaccinations [54]. These findings highlight the necessity of focused, multifaceted public health measures to rectify misconceptions, encourage complementary immunization procedures, and sustain balanced communication regarding various infection risks.

This review identified several critical gaps in the current evidence base. First, there has been relatively limited exploration of practical issues [83]. Although recent initiatives, such as the “pay-it-forward” program, have begun addressing economic and social challenges, a thorough investigation of practical barriers across various contexts remains inadequate. Further study is required to understand how different healthcare financing models (e.g., universal health coverage, out-of-pocket payments) and alternative forms of financing specifically impact older individuals in HICs, MICs, and particularly LICs contexts. Second, the correlation between vaccination intentions and actual vaccination behaviors is inadequately investigated, as most studies employ cross-sectional designs that do not consider temporal changes or causative factors. Longitudinal studies are essential for understanding the dynamics of intention and behavior, particularly in differentiating between rational decision-making and habitual vaccination behaviors among older adults. This is especially relevant in the context of significant health events or policy changes that may impact these patterns [84]. Third, there is a marked inequality in studies conducted in LICs. The majority of evidence from HICs and MICs constrains the global generalizability of findings and impedes the implementation of equitable vaccination strategies [30,75]. The variation in factors influencing influenza vaccination between HICs and MICs, which is shaped by disparities in healthcare systems, vaccination policies, and socioeconomic contexts, underscores the potential risks of generalizing study findings to LICs. Future research should prioritize low-income populations by addressing their distinct healthcare infrastructure, sociocultural context, and access barriers. Fourth, comparative studies specifically differentiating older individuals from the overall adult population are still limited. These studies may clarify age-specific factors, hence enhancing the efficacy of adapted interventions. Fifth, this review examined the impact of the COVID-19 pandemic on influenza vaccination; however, the pandemic-related aspects affecting older persons require further research. These include the potential impact of the COVID-19 vaccine mandate on subsequent attitudes and behaviors toward influenza vaccination, as well as the extent to which public concerns about adverse events associated with the vaccine may have influenced perceptions and acceptance of influenza vaccines in this population. Addressing these gaps requires interdisciplinary collaboration, culturally sensitive methodologies, and sustained investment in longitudinal, comparative, and system-level studies.

This review has several strengths. First, we systematically applied the BeSD framework to synthesize evidence on influenza vaccine uptake in the elderly, providing a structured and comprehensive understanding across all relevant domains. Second, the inclusion of studies from 24 countries enhances the geographic diversity and generalizability of the findings. Third, adherence to rigorous methodological standards, including the use of the JBI methodology and reporting according to the PRISMA-ScR guidelines, reinforces the robustness, transparency, and reproducibility of the review process. Fourth, by applying the BeSD model specifically to the elderly and considering the potential integration of findings with evidence from the general adult population, this review provides a more refined understanding of age-specific behavioral and social determinants of influenza vaccination.

Nevertheless, certain limitations should be acknowledged. First, the restriction to English-language publications may have introduced language bias, potentially excluding relevant studies published in other languages. Second, the high number of cross-sectional study designs among the included studies restricts causal inference and the ability to capture behavioral trajectories over time. Third, considerable variation in study designs, populations, healthcare systems, and outcome measures may diminish the comparability of study results and constrain the applicability of direct synthesis or replication. Fourth, the restricted representation of studies from LICs limits the applicability of findings to resource-constrained settings. Fifth, although attempts have been made to compare behavioral drivers between older individuals and the broader population, such comparisons are interpretative and constrained by a lack of research that has included both demographics in similar settings. Despite these limitations, the breadth and depth of the evidence synthesized in this review provide valuable insights to inform public health policy, guide targeted interventions, and promote equitable influenza vaccine uptake among older adults globally.

## 5. Conclusions

This scoping review synthesized behavioral and social drivers of influenza vaccination among older adults within the WHO BeSD framework. By mapping facilitators and barriers across psychological, social, motivational, and practical domains, the review highlights key factors contributing to suboptimal vaccine uptake. The findings underscore the need for behaviorally informed, equity-oriented strategies that address both individual-level determinants and broader structural constraints. Particular attention needs to be paid to areas that have not yet been fully explored, such as cultural alignment issues that the BeSD framework may not address, the multifaceted practical barriers faced by older adults, and the complex intention–behavior gaps that may emerge in the post-COVID-19 era.

## Figures and Tables

**Table 1:** Summary of included studies (*n* = 39).

Study	Year Country (Region)	Study Type	Study Population and Sample Size	Study Timeline
**High Income Countries (HIC)**				
Nexøe J et al. [22]	1999 Demark (National)	Quantitative	1775 individuals aged ≥ 65 years	1996 to 1997
Bosompra K et al. [23]	2004 USA (Vermont)	Quantitative	799 individuals aged ≥ 65 years	2000
Zimmerman K et al. [24]	2004 USA (Pennsylvania)	Quantitative	925 individuals aged ≥ 65 years	2000
Adonis-Rizzo T et al. [25]	2007 USA (Florida)	Qualitative	10 individuals aged ≥ 60 years	2005 to 2006
Anne-Laure B et al. [26]	2014 France (National)	Quantitative	269 individuals aged ≥ 65 years	2009 to 2010
Bodekers B et al. [27]	2015 Germany (National)	Quantitative	760 individuals aged ≥ 65 years	2014
Dyda A et al. [28]	2015 Australia (New South Wales)	Quantitative	7332 individuals aged ≥ 65 years	2006 to 2008
Klett-Tammen J et al. [29]	2016 Germany (National)	Quantitative	1223 individuals aged ≥ 60 years	2012 to 2015
Ganczak M et al. [30]	2017 Poland (Szczecin)	Quantitative	230 individuals aged ≥ 65 years	2015 to 2016
Rikin S et al. [31]	2018 USA (New York)	Mixed-Methods	200 Hispanic individuals aged ≥ 65 years	2016
Kajikawa N et al. [32]	2019 Japan (Ibaraki)	Quantitative	316 persons aged above 65 years	2018
Teo M et al. [33]	2019 Singapore (National)	Qualitative	15 individuals aged ≥ 65 years	2017
Dardalas I et al. [34]	2020 Greece (4 Regions)	Quantitative	318 individuals aged ≥ 60 years	2018
Nicholls B et al. [9]	2021 UK (National)	Quantitative	372 individuals aged ≥ 65 years	2020
Pietraszek A et al. [35]	2022 Poland (National)	Quantitative	500 individuals aged ≥ 60 years	2020
Music M et al. [36]	2023 Canada (Ontrario)	Mixed-Methods	33 individuals aged ≥ 65 years	2021 to 2022
Xu Y et al. [37]	2023 Singapore (National)	Mixed-Methods	235 individuals aged ≥ 65 years	2020 to 2021
Fuller R et al. [8]	2024 USA (North Dakota)	Quantitative	901 individuals aged ≥ 65 years	2022
**Middle Income Countries (MIC)**				
Kwong Y et al. [38]	2009 China (Hongkong)	Quantitative	197 individuals aged ≥ 65 years	2005
Victor F et al. [39]	2014 Brazil (Fortaleza)	Quantitative	286 individuals aged ≥ 60 years	2010 to 2011
Yu S et al. [40]	2014 China (Hongkong)	Quantitative	306 individuals aged ≥ 65 years	2011
Mo H et al. [41]	2015 China (Hongkong)	Quantitative	1101 individuals aged ≥ 65 years	2009
Praphasiri P et al. [42]	2017 Thailand (Nakhon Phano)	Quantitative	581 individuals aged ≥ 65 years	2014
Wu S et al. [43]	2017 China (Beijing)	Quantitative	1362 individuals aged ≥ 60 years	2015
Ye C et al. [44]	2018 China (Shanghai)	Quantitative	4417 individuals aged ≥ 60 years	2016 to 2017
Gazibara T et al. [45]	2019 Serbia (Belgrade)	Quantitative	480 individuals aged ≥ 65 years	2012 to 2013
Jiang X et al. [46]	2020 China (Zhejiang)	Quantitative	1210 individuals aged ≥ 60 years	2019
Kizmaz M et al. [47]	2020 Turkey (Gemerek)	Quantitative	326 individuals aged ≥ 65 years	2019
Zhang F et al. [48]	2020 China (Hongkong)	Quantitative	486 individuals aged ≥ 65 years	2016
Kharroubi G et al. [49]	2021 Tunisia (National)	Quantitative	1191 individuals aged ≥ 60 years with chronic disease	2019
Siu Y et al. [50]	2021 China (Hongkong)	Qualitative	40 individuals aged ≥ 65 years	2016
Zhou Y et al. [51]	2021 China (Shanghai)	Quantitative	445 individuals aged ≥ 60 years	2020
Che X et al. [52]	2022 China (Hangzhou)	Quantitative	11,663 individuals aged ≥ 70 years	2022
Hou Z et al. [53]	2022 China (National)	Quantitative	3849 individuals aged ≥ 60 years	2019
You Y et al. [54]	2023 China (Shenzhen)	Quantitative	975 individuals aged ≥ 60 years	2021
Shen Y et al. [55]	2024 China (Beijing)	Quantitative	540 individuals aged ≥ 65 years	2016 to 2020
Zhao Z et al. [56]	2024 China (Guangdong)	Quantitative	423 individuals aged ≥ 60 years	2023
**Mixed**				
Kwong Y et al. [57]	2010 Multiple	Qualitative	208 individuals aged ≥ 65 years	2006 to 2007
Schulz J et al. [58]	2019 Multiple	Quantitative	627 individuals aged ≥ 65 years	2017

Note. Studies are grouped by country income level (High-Income Countries [HICs], Middle-Income Countries [MICs], and Mixed) based on World Bank income groupings. Within each group, studies are ordered by year of publication, and those from the same year are sorted alphabetically by the first author’s surname.

**Table 2:** Mapping of Extracted BeSD Domains and Related Variables (*n* = 39).

Study	BeSD Domains				Theoretical Model	Sociodemographic Characteristics	Health-Related Factors	Outcomes
	Thinking and Feeling	Social Process	Motivation	Practical Issues				
**High Income Countries (HIC)**								
Nexøe J et al., 1999 [22]	* Perceived benefits, Perceived severity # Perceived barriers				Health belief model, Multidimensional health locus of control	Age, Living status, Residence	Influenza vaccination history	Influenza vaccination behavior
Bosompra K et al., 2004 [23]	* Perceived benefits, Normative beliefs about vaccination				The theory of reasoned action			Influenza vaccination behavior
Zimmerman K et al., 2004 [24]	* Risk perception	* Recommendation from HCWs	* Vaccination intention		The theory of reasoned action	Residence	Cancer screening history	Influenza vaccination behavior
Adonis-Rizzo T et al., 2007 [25]	* Fear of illness	* Recommendation from HCWs		# Lack of insurance, Transportation and geographic barriers, Language barriers ^	Health belief model			Influenza vaccination behavior or intention
Anne-Laure B et al., 2014 [26]		* Social trust						Influenza vaccination behavior
Bodekers B et al., 2015 [27]	* Risk Perception, Positive vaccine attitude # Fear of side effects	* Recommendation from HCWs				Gender, Age	Chronic diseases	Influenza vaccination behavior
Dyda A et al., 2015 [28]				* Free cost # Language and cultural barriers ^		Age, Sex, Income, Education level, Country, Region, Career status	Obesity, Physical assistance need, Chronic diseases	Influenza vaccination behavior
Klett-Tammen J et al., 2016 [29]	* Vaccine confidence	* Recommendation from HCWs						Influenza vaccination behavior
Ganczak M et al., 2017 [30]		* Family influence, Information provision	* Vaccination intention			Age	Health status, Comorbidities	Influenza vaccination behavior
Rikin S et al., 2018 [31]	# Fear of side effects							Influenza vaccination behavior
Kajikawa N et al., 2019 [32]	* Vaccine confidence	* Recommendation from HCWs				Age		Influenza vaccination behavior
Teo M et al., 2019 [33]	# Fear of side effects, Perceived low risk	* Recommendation from HCWs, Recommendation from Family, Recommendation from Friends						Influenza vaccination intention
Dardalas I et al., 2020 [34]	* Behavioral beliefs, Control beliefs	* Family influence, Peer influence	* Vaccination intention		Theory of planned behavior	Gender		Influenza vaccination behavior and intention
Nicholls B et al., 2021 [9]	* Risk perception, Preference for natural immunity ^		* Collective Responsibility					Influenza vaccination behavior
Pietraszek A et al., 2022 [35]	* Risk Perception (Context: COVID-19 pandemic)					Income	Chronic disease	Influenza vaccination behavior
Music M et al., 2023 [36]	* Vaccine confidence	* Social trust, Information provision # Misinformation	# Vaccine hesitancy					Influenza vaccination behavior
Xu Y et al., 2023 [37]	* Risk perception (Context: COVID-19 pandemic) # Fear of side effects, Lack of vaccine confidence		* Collective responsibility	# High cost		Living status		Influenza vaccination behavior
Fuller R et al., 2024 [8]	# Fear of injections	# Lack of recommendation from HCWs		# High costs, Accessibility barriers		Residence, Educational level, Ethnicity, Living conditions		Influenza vaccination behavior within the previous 5 years
**Middle Income Countries (MIC)**								
Kwong Y et al., 2009 [38]	* Perceived susceptibility, Perceived severity, Perceived benefits # Perceived barriers	* Recommendation from HCWs, Recommendation from family			Health belief model			Influenza vaccination behavior
Victor F et al., 2014 [39]	* Perceived benefits	* Recommendation from HCWs		* Information accessibility		Age	Chronic disease, Medication use, Regular health monitoring, Frequency of visits to FHCs, Participation in FHC groups	Influenza vaccination behavior
Yu S et al., 2014 [40]	* Perceived susceptibility			# Postvaccination discomfort				Influenza vaccination intention
Mo H et al., 2015 [41]	* Risk perception, Fear of side effects	* Recommendation from HCWs, Community engagement		* Low cost		Age	Chronic diseases	Past influenza vaccination behavior, Influenza vaccination intention
Praphasiri P et al., 2017 [42]	* Vaccine confidence	* Recommendation from HCWs		# Accessibility barriers		Fragile		Influenza vaccination behavior
Wu S et al., 2017 [43]	* Risk perception # Fear of side effects	* Recommendation from HCWs		* Free cost		Education level	Chronic disease	Influenza vaccination behavior
Ye C et al., 2018 [44]	# Low vaccine literacy					Living status		Influenza vaccination behavior
Gazibara T et al., 2019 [45]	* Vaccine literacy, Vaccine confidence # Fear of side effects, Perceived low risk	* Recommendation from HCWs, Family influence				Educational level		Influenza vaccination behavior
Jiang X et al., 2020 [46]	* Vaccine literacy, Risk Perception, Vaccine confidence			* Free cost		Age, Education level, Income, Family construct	Health status	Influenza vaccination behavior
Kizmaz M et al., 2020 [47]		* Recommendation from HCWs						Influenza vaccination behavior
Zhang F et al., 2020 [48]	* Vaccine literacy # Cognitive overload, Decisional conflict			# Limited information access		Age, Education level	Health condition	Influenza vaccination behavior
Kharroubi G et al., 2021 [49]	* Vaccine literacy, Vaccine confidence # Fear of side effects							Past influenza vaccination behavior, Influenza vaccination intention
Siu Y et al., 2021 [50]	# Negative vaccine beliefs, low perceived susceptibility, Perceived risk posed by the vaccination locations ^, Stereotypes of hospitals and clinics ^	# Misinformation, Limited provider engagement			Health belief model			Influenza vaccination decision-making processes
Zhou Y et al., 2021 [51]			* Vaccination Intention (Context: COVID-19 pandemic)	* Free cost				Influenza vaccination behavior
Che X et al., 2022 [52]	* Vaccine literacy	* Social trust		* Vaccination convenience		Age, Education level, Region		Influenza vaccination intention
Hou Z et al., 2022 [53]	* Risk perception, Vaccine confidence # Fear of side effects		# Vaccine hesitancy	* Free cost	3Cs Model	Gender, Educational level, Residence		Influenza vaccination behavior
You Y et al., 2023 [54]	* Vaccine Confidence, Vaccine literacy					Age, Marital status, Education level, Income	COVID-19 vaccination history	Influenza vaccination behavior
Shen Y et al., 2024 [55]	* Vaccine confidence # Perceived low risk			# Accessibility barriers		Age, Income, Residence	Chronic disease	Influenza vaccination behavior within 5 years
Zhao Z et al., 2024 [56]	* Positive vaccine attitude, Risk perception # Low vaccine literacy, Perceived cost barriers	* Social trust	# Vaccine hesitancy			Income, Rural area		Influenza vaccination behavior
**Mixed**								
Kwong Y et al., 2010 [57]	* Perceived risk, Vaccine confidence # Fear of side effects, Fear of injections, Preference for natural immunity ^	* Recommendation from HCWs, Family influence, Peer influence, Community norms	* Vaccination intention, Moral motivation	# High cost, Accessibility issues, Vaccine availability shortages, Health system barriers (e.g., insurance coverage, ease of access)	Health belief model		Health condition, Chronic disease, Traditional health practices	Influenza vaccination behavior
Schulz J et al., 2019 [58]		* Recommendation from HCWs, Recommendation from Family, Recommendation from Friends				Age, Sex		Influenza vaccination behavior

Note: “*” indicates facilitators; “#” indicates barriers; “^” indicates factors related to cultural alignment. BeSD = Behavioral and Social Drivers; HCWs = Health Care Workers; FHCs = Family Health Centers. Studies are grouped and ordered using the same structure as in Table 1: by country income level, year of publication, and alphabetical order by first author within each year.

## Data Availability

No new data were created or analyzed in this study; therefore, data sharing does not apply to this article.

## Appendix A

### Appendix A.1. Understanding Behavioural and Social Drivers in Influenza Vaccination in Older Adults—Protocol

**Background**

Influenza vaccination is vital for healthy aging, yet coverage among older adults remains below the WHO’s 75% target in many countries. The WHO’s Behavioral and Social Drivers (BeSD) framework offers a comprehensive lens to identify factors influencing vaccine uptake: thinking and feeling, social processes, motivation, and practical issues. This scoping review synthesizes current evidence on these drivers among adults aged 60 and older to inform interventions aimed at boosting vaccine coverage.

**Central question**

This scoping review aims to address the following central question: What are the behavioral and social drivers associated with influenza vaccination uptake among older adults aged 60 years and older?

**Review objectives**

The objectives of this scoping review are to attain the following: (a) identify the behavioral and social factors that influence influenza vaccination among older adults aged 60 years and above; (b) classify facilitators and barriers to vaccination according to the WHO Behavioral and Social Drivers (BeSD) framework; and (c) identify knowledge gaps in the current evidence base to inform future research and intervention strategies.

**Review Methods**

Justification

A scoping review methodology is appropriate for mapping the existing literature on behavioral and social drivers of influenza vaccination among older adults. This approach allows for a comprehensive overview of the available evidence, helps clarify key concepts, and identifies research gaps. Given the emerging and complex nature of this topic, a scoping review will provide the necessary breadth without restricting the findings to narrowly defined questions as required in a systematic review.

Inclusion and exclusion criteria

- Population: The population of interest is older adults aged 60 years and older. Studies with mixed-age populations will be included if results for the older adult subgroup are reported separately.
- Concept: The review will focus on behavioral and social drivers related to influenza vaccination. Eligible studies must address at least one domain of the BeSD framework, namely Thinking and Feeling, Social Processes, Motivation, or Practical Issues.
- Context: There are no restrictions on geographic location or healthcare setting. Studies conducted in community, clinical, or institutional settings will be eligible.
- Research detail:
  (a) Language: only studies published in English will be included;
  (b) Published Time: studies published up to 31 December 2024 will be considered;
  (c) Study method: all study designs, including qualitative, quantitative, and mixed methods studies, will be included;
  (d) Study Type Exclusion: Clinical reports, guidelines, position papers, study protocols, book chapters, conference abstracts, editorials, duplicate studies, and studies without full text will be excluded;
  (e) Topical Exclusion: Studies focusing solely on vaccine properties (e.g., efficacy, safety, immunogenicity), cost-effectiveness analyses, modeling studies, or budget impact analyses will be excluded. Studies focusing on vaccines unrelated to influenza vaccination will also be excluded.

Search strategy

- Database

A comprehensive search will be conducted using the following databases: MEDLINE (via PubMed), EMBASE, Web of Science, CINAHL, and Cochrane Library.

- Search keyword

The search will combine terms related to:

(a) Population: “older adults”, “elderly”, “aged 60 years and older”
(b) Intervention: “influenza vaccination”, “flu shot”, “immunization”
(c) Behavioral and social drivers: “perception”, “acceptance”, “motivation”, “willingness”, “intention”, “hesitancy”, “social norms”, “recommendations”, “trust”, “barriers”, “facilitators”

- Search steps

All search results will be imported into EndNote 21 for management. Two reviewers will independently screen the titles and abstracts against the eligibility criteria. Full texts of potentially eligible articles will be retrieved and assessed independently. Discrepancies between reviewers will be resolved through discussion, and if consensus cannot be reached, a third reviewer will adjudicate.

**Data extraction**

We will conduct the review according to PRISMA (Preferred Reporting Items for Systematic Reviews and Meta-Analyses) guidelines. Data from included reviews will be extracted by one reviewer and independently checked by a second reviewer. We will extract the basic information of the included studies and the key issues, and the data extraction table will be provided later.

### Appendix A.2. PRISMA-ScR Checklist

**SECTION**	**ITEM**	**PRISMA-ScR CHECKLIST ITEM**	**REPORTED ON PAGE #**
**TITLE**			
Title	1	Identify the report as a scoping review.	1
**ABSTRACT**			
Structured summary	2	Provide a structured summary that includes (as applicable) the following: background, objectives, eligibility criteria, sources of evidence, charting methods, results, and conclusions that relate to the review questions and objectives.	1
**INTRODUCTION**			
Rationale	3	Describe the rationale for the review in the context of what is already known. Explain why the review questions/objectives lend themselves to a scoping review approach.	1–2
Objectives	4	Provide an explicit statement of the questions and objectives being addressed with reference to their key elements (e.g., population or participants, concepts, and context) or other relevant key elements used to conceptualize the review questions and/or objectives.	2
**METHODS**			
Protocol and registration	5	Indicate whether a review protocol exists; state if and where it can be accessed (e.g., a Web address); and, if available, provide registration information, including the registration number.	2 and Appendix A.1
Eligibility criteria	6	Specify characteristics of the sources of evidence used as eligibility criteria (e.g., years considered, language, and publication status) and provide a rationale.	3
Information sources	7	Describe all information sources in the search (e.g., databases with dates of coverage and contact with authors to identify additional sources), as well as the date the most recent search was executed.	3 and Appendix A.3
Search	8	Present the full electronic search strategy for at least one database, including any limits used, such that it could be repeated.	3 and Appendix A.3
Selection of sources of evidence	9	State the process for selecting sources of evidence (i.e., screening and eligibility) included in the scoping review.	3
Data charting process	10	Describe the methods of charting data from the included sources of evidence (e.g., calibrated forms or forms that have been tested by the team before their use and whether data charting was conducted independently or in duplicate) and any processes for obtaining and confirming data from investigators.	3
Data items	11	List and define all variables for which data were sought and any assumptions and simplifications made.	3
Critical appraisal of individual sources of evidence	12	If conducted, provide a rationale for conducting a critical appraisal of included sources of evidence; describe the methods used and how this information was used in any data synthesis (if appropriate).	3-4 and Appendix A.4
Synthesis of results	13	Describe the methods of handling and summarizing the data that were charted.	4
**RESULTS**			
Selection of sources of evidence	14	Give the number of sources of evidence screened, assessed for eligibility, and included in the review, with reasons for exclusions at each stage, ideally using a flow diagram.	4 and Figure 1
Characteristics of sources of evidence	15	For each source of evidence, present characteristics for which data were charted and provide the citations.	4–5 and Table 1
Critical appraisal within sources of evidence	16	If conducted, present data on critical appraisal of included sources of evidence (see item 12).	Appendix A.4
Results of individual sources of evidence	17	For each included source of evidence, present the relevant data that were charted that relate to the review questions and objectives.	5 and Table 2
Synthesis of results	18	Summarize and/or present the charting results as they relate to the review questions and objectives.	13–14
**DISCUSSION**			
Summary of evidence	19	Summarize the main results (including an overview of concepts, themes, and types of evidence available), link to the review questions and objectives, and consider the relevance to key groups.	14
Limitations	20	Discuss the limitations of the scoping review process.	17
Conclusions	21	Provide a general interpretation of the results with respect to the review questions and objectives, as well as potential implications and/or next steps.	17–18
**FUNDING**			
Funding	22	Describe sources of funding for the included sources of evidence, as well as sources of funding for the scoping review. Describe the role of the funders of the scoping review.	18

### Appendix A.3. Search Strategy and Results

1. MEDLINE Search Strategy

Database: MEDLINE via PubMed

Search Date: 18 February 2025

**#**	**Searches**	**Results**
1	‘Influenza, Human’ [Mesh] OR flu[Title/Abstract] OR influenza[Title/Abstract] OR grippe[Title/Abstract]	142,111
2	Vaccines[Mesh] OR vaccin*[Title/Abstract] OR immuniz*[Title/Abstract] OR immunis*[Title/Abstract] OR inoculat*[Title/Abstract] OR shot*[Title/Abstract]	748,464
3	aged[Mesh] OR ‘old people’[Title/Abstract] OR ‘older people’[Title/Abstract] OR ‘old adults’[Title/Abstract] OR ‘old age’[Title/Abstract] OR aging[Title/Abstract] OR elder[Title/Abstract] OR elderly[Title/Abstract] OR senior[Title/Abstract] OR seniors[Title/Abstract] OR geriatric[Title/Abstract]	3,987,173
4	percept*[Title/Abstract] OR accept*[Title/Abstract] OR motivat*[Title/Abstract] OR refus*[Title/Abstract] OR decision*[Title/Abstract] OR attitude[Title/Abstract] OR intention*[Title/Abstract] OR uptake [Title/Abstract] OR knowledge[Title/Abstract] OR willingness[Title/Abstract] OR unwilling*[Title/Abstract] OR adherence[Title/Abstract] OR compliance[Title/Abstract] obedience[Title/Abstract] OR support[Title/Abstract] OR motivation[Title/Abstract] OR consideration[Title/Abstract]	1,693,779
	1 AND 2 AND 3 AND 4	569
	Filters: Humans, English, from the database set—2024	525
* Truncation symbol used in search strings to substitute for zero or more characters at the end of a word.		

2. Embase Search Strategy

Database: Embase via Elsevier

Search Date: 2 February 2025

**#**	**Searches**	**Results**
1	‘influenza‘/exp OR influenza*:ab,ti OR flu:ab,ti OR grippe:ab,ti	245,497
2	‘vaccine‘/exp OR ‘vaccination‘/exp OR vaccin*:ab,ti OR immuniz*:ab,ti OR immunis*:ab,ti OR incoculat*:ab,ti OR shot*:ab,ti	845,190
3	‘aged‘/exp OR ‘old people‘:ab,ti OR ‘older people‘:ab,ti OR ‘older adults‘:ab,ti OR ‘old age‘:ab,ti OR aging:ab,ti OR elder:ab,ti OR elderly:ab,ti OR senior:ab,ti OR seniors:ab,ti OR geriatric:ab,ti	4,617,288
4	percept*:ab,ti OR accept*:ab,ti OR motivat*:ab,ti OR refus*:ab,ti OR decision*:ab,ti OR attitude:ab,ti OR intention*:ab,ti OR uptake:ab,ti OR knowledge:ab,ti OR willingness:ab,ti OR unwilling*:ab,ti OR adherence:ab,ti OR compliance:ab,ti OR obedience:ab,ti OR support:ab,ti OR motivation:ab,ti OR consideration:ab,ti	6,016,676
	1 AND 2 AND 3 AND 4	3476
	AND [article]/lim AND [english]/lim AND ‘human’/de AND [embase]/lim AND (2014:py OR 2015:py OR 2016:py OR 2017:py OR 2018:py OR 2019:py OR 2020:py OR 2021:py OR 2022:py OR 2023:py OR 2024:py)	2353
* Truncation symbol used in search strings to substitute for zero or more characters at the end of a word.		

3. Web of Science Search Strategy

Database: Web of Science Core Collection Citation Indexes Clarivate

Search Date: 2 February 2025

**#**	**Search**	**Results**
1	TS = (‘influenza, human’ OR ‘influenza*’ OR ‘flu’ OR ‘grippe’)	198,337
2	TS = (‘vaccin*’ OR ‘immuniz*’ OR ‘immunis*’ OR ‘incoculat*’ OR ‘shot*)	750,003
3	TS = (‘aged’ OR ‘old people’ OR ‘older people’ OR ‘older adults’ OR ‘old age’ OR ‘aging’ OR ‘elder’ OR ‘elderly’ OR ‘senior’ OR ‘seniors’ OR ‘geriatric’)	6,579,376
4	TS = (‘percept*’ OR ‘accept*’ OR ‘motivat*’ OR ‘refus*’ OR ‘decision’ OR ‘attitude’ OR ‘intention’ OR ‘uptake’ OR ‘knowledge’ OR ‘willingness’ OR ‘unwilling’ OR ‘adherence’ OR ‘compliance’ OR ‘obedience’ OR ‘support’ OR ‘motivation’ OR ‘consideration’)	11,812,077
	1 AND 2 AND 3 AND 4	6590
	AND 2025 (Exclude—Publication Years) and Article (Document Types) and English (Languages) and Article (Document Types) and Proceeding Paper or Early Access or Book Chapters (Exclude—Document Types)	4760
* Truncation symbol used in search strings to substitute for zero or more characters at the end of a word.		

4. CINAHL Search Strategy

Database: CINAHL (Cumulative Index of Nursing and Allied Health Literature via EBSCO)

Search Date: 2 February 2025

**#**	**Search**	**Results**
1	MH influenza, human OR TI influenza OR AB influenza OR TI flu OR AB flu OR TI grippe OR AB grippe	29,051
2	MH vaccines+ OR MH immunization+ OR TI vaccin* OR AB vaccin* OR TI immuniz* OR AB immuniz* OR TI immunis* OR AB immunis* OR TI inoculat* OR AB inoculat* OR TI shot* OR AB shot*	115,737
3	MH aged OR TI old people OR AB old people OR TI older people OR AB older people OR TI old adults OR AB old adults OR TI old age OR AB old age OR TI aging OR AB aging OR TI elder OR AB elder OR TI elderly OR AB elderly OR TI senior OR AB senior OR TI seniors OR AB seniors OR TI geriatric OR AB geriatric	1,066,273
4	TI (percept* OR accept* OR motivat* OR refus* OR decision OR attitude OR intention OR uptake OR knowledge OR willingness OR unwilling OR adherence OR compliance OR obedience OR support OR motivation OR consideration) OR AB (percept* OR accept* OR motivat* OR refus* OR decision OR attitude OR intention OR uptake OR knowledge OR willingness OR unwilling OR adherence OR compliance OR obedience OR support OR motivation OR consideration)	1,344,905
	1 AND 2 AND 3 AND 4	880
	Limiters—Date Published: 20241231; English Language; Research Article; Human	658
* Truncation symbol used in search strings to substitute for zero or more characters at the end of a word.		

5. Cochrane Library Search Strategy

Database: Cochrane Library

Search Date: 2 February 2025

**#**	**Search**	**Results**
1	MeSH descriptor: [Influenza, Human] explode all trees	3703
2	(influenza* OR flu OR grippe):ti,ab,kw	13,659
3	1 OR 2	13,659
4	MeSH descriptor: [Vaccines] in all MeSH products	17,900
5	(vaccin* OR immuniz* OR immunis* OR incoculat* OR shot*):ti,ab,kw	39,909
6	4 OR 5	39,931
7	MeSH descriptor: [Aged] in all MeSH products	285,395
8	(‘old people’ OR ‘older people’ OR ‘old adults’ OR ‘old age’ OR ‘aging’ OR ‘elder’ OR ‘elderly’ OR ‘senior’ OR ‘seniors’ OR ‘geriatric’):ti,ab,kw	185,408
9	#7 OR #8	425,146
10	(percept* OR accept* OR motivat* OR refus* OR decision OR attitude OR intention OR uptake OR knowledge OR willingness OR unwilling OR adherence OR compliance OR obedience OR support OR motivation OR consideration):ti,ab,kw	464,591
11	#3 AND #6 AND #9 AND #10	620
	with Publication Year to 2024, with Cochrane Library publication date to Dec 2024, in Trials	606
* Truncation symbol used in search strings to substitute for zero or more characters at the end of a word.		

### Appendix A.4. Critical Appraisal Checklist

**Table A1:** JBI Critical Appraisal Checklist for analytical cross-sectional studies in 33 articles about vaccine coverage data.

Studies	Were the Criteria for Inclusion in the Sample Clearly Defined?	Were the Study Subjects and the Setting Described in Detail?	Was the Exposure Measured in a Valid and Reliable Way?	Were Objective and Standard Criteria Used for Measurement of the Condition?	Were Confounding Factors Identified?	Were Strategies to Deal with Confounding Factors Stated?	Were the Outcomes Measured in a Valid and Reliable Way?	Was Appropriate Statistical Analysis Used?
Anne-Laure B et al. [26]	Yes	Yes	Yes	Yes	Yes	Yes	Yes	Yes
Bodekers B et al. [27]	Yes	Yes	Yes	Yes	Yes	Yes	Yes	Yes
Bosompra K et al. [23]	Yes	Yes	Yes	Unclear	No	No	Yes	Yes
Dardalas I et al. [34]	Yes	Yes	Yes	Yes	Yes	Yes	Yes	Yes
Dyda A et al. [28]	Yes	Yes	Yes	Yes	Yes	Yes	Yes	Yes
Fuller R et al. [8]	Yes	Yes	Yes	Yes	Yes	Yes	Yes	Yes
Ganczak M et al. [30]	Yes	Yes	Yes	Yes	Yes	Yes	Yes	Yes
Kaijikawa N et al. [32]	Yes	Yes	Yes	Yes	Yes	Unclear	Yes	Yes
Klett-Tammen J et al. [29]	Yes	Yes	Yes	Yes	Yes	Yes	Yes	Yes
Nexøe J et al. [22]	Yes	Yes	Yes	Yes	Yes	Unclear	Yes	Yes
Nicholls B et al. [9]	Yes	Yes	Yes	Yes	Yes	Unclear	Yes	Yes
Pietraszek A et al. [35]	Yes	Yes	Yes	Yes	Yes	Unclear	Yes	Yes
Zimmerman K et al. [24]	Yes	Yes	Yes	Yes	Yes	Yes	Yes	Yes
Che X et al. [52]	Yes	Yes	Yes	Yes	Yes	Yes	Yes	Yes
Gazibara T et al. [45]	Yes	Yes	Yes	Yes	Unclear	Yes	Yes	Yes
Hou Z et al. [53]	Yes	Yes	Yes	Yes	Yes	Yes	Yes	Yes
Jiang X et al. [46]	Yes	Yes	Yes	Yes	Yes	Yes	Yes	Yes
Kharroubi G et al. [49]	Yes	Yes	Yes	Yes	Yes	Yes	Yes	Yes
Kizmaz M et al. [47]	Yes	Yes	Yes	Yes	Yes	Unclear	Yes	Yes
Kwong Y. et al. [38]	Yes	Yes	Yes	Yes	Yes	Yes	Yes	Yes
Mo H et al. [41]	Yes	Yes	Yes	Yes	Yes	Unclear	Yes	Yes
Praphasiri P et al. [42]	Yes	Yes	Yes	Yes	Yes	Yes	Yes	Yes
Shen Y et al. [55]	Yes	Yes	Yes	Yes	Yes	Yes	Yes	Yes
Victor F et al. [39]	Yes	Yes	Yes	Yes	Unclear	Yes	Yes	Yes
Wu S et al. [43]	Yes	Yes	Yes	Yes	Yes	Yes	Yes	Yes
Ye C et al. [44]	Yes	Yes	Yes	Yes	Yes	Unclear	Yes	Yes
You Y et al. [54]	Yes	Yes	Yes	Yes	Yes	Yes	Yes	Yes
Yu S et al. [40]	Yes	Yes	Yes	Yes	Yes	Unclear	Yes	Yes
Zhang F et al. [48]	Yes	Yes	Yes	Yes	Yes	Yes	Yes	Yes
Zhao Z et al. [56]	Yes	Yes	Yes	Yes	Yes	Yes	Yes	Yes
Zhou Y et al. [51]	Yes	Yes	Yes	Yes	Unclear	Yes	Yes	Yes
Schulz J et al. [58]	Yes	Yes	Yes	Yes	Yes	Unclear	Yes	Yes

**Table A2:** JBI Critical Appraisal Checklist for qualitative research in the four articles.

Studies	Is There Congruity Between the Stated Philosophical Perspective and the Research Methodology?	Is There Congruity Between the Research Methodology and the Research Question or Objectives?	Is There Congruity Between the Research Methodology and the Methods Used to Collect Data?	Is There Congruity Between the Research Methodology and the Representation and Analysis of Data?	Is There Congruity Between the Research Methodology and the Interpretation of Results?	Are Participants and Their Voices Adequately Represented?	Is the Research Ethical According to Current Criteria or for Recent Studies, and Is There Evidence of Ethical Approval by an Appropriate Body?	Were the Conclusions Drawn in the Research Report Flow from the Analysis or Interpretation of the Data?
Adonis-Rizzo T et al. [25]	Yes	Yes	Yes	Yes	Yes	Yes	Yes	Yes
Teo M et al. [33]	Yes	Yes	Yes	Yes	Yes	Yes	Yes	Yes
Siu Y et al. [50]	Yes	Yes	Yes	Yes	Yes	Yes	Yes	Yes
Kwong Y et al. [57]	Yes	Yes	Yes	Yes	Yes	Yes	Yes	Yes

**Table A3:** MMAT 2018 version for mixed methods research in the three articles.

Studies	Are There Clear Research Questions?	Do the Collected Data Allow Addressing the Research Questions?	Is There an Adequate Rationale for Using a Mixed Methods Design to Address the Research Question?	Are the Different Components of the Study Effectively Integrated to Answer the Research Question?	Are the Outputs of Integration of Qualitative and Quantitative Components Adequately Interpreted?	Are Divergences and Inconsistencies Between Quantitative and Qualitative Results Adequately Addressed?	Do the Different Components of the Study Adhere to the Quality Criteria of Each Tradition of the Methods Involved?
Music M. et al. [36]	Yes	Yes	Yes	Yes	Yes	Cannot tell	Yes
Rikin S et al. [31]	Yes	Yes	Yes	Yes	Yes	Cannot tell	Yes
Xu Y et al. [37]	Yes	Yes	Yes	Yes	Yes	Cannot tell	Yes
